# Supplementary material for: Effective electrical manipulation of a topological antiferromagnet by orbital torques
Source: Nat Commun. 2024 Jan 25;15:745. doi: 10.1038/s41467-024-45109-1 (PMC10811228; doi:10.1038/s41467-024-45109-1)
Supplement: Supplementary file 1 — Supplementary Information [file 41467_2024_45109_MOESM1_ESM.pdf]

Supplementary Materials for

Effective electrical manipulation of a topological antiferromagnet by orbital torques

Zhenyi Zheng[1,†], Tao Zeng[1,†], Tieyang Zhao[1,†], Shu Shi[1], Lizhu Ren[2], Tongtong Zhang[3], Lanxin Jia[1], Youdi Gu[1], Rui Xiao[1], Hengan Zhou[1], Qihan Zhang[1], Jiaqi Lu[1], Guilei Wang[4], Chao Zhao[4], Huihui Li[4*], Beng Kang Tay[3*], Jingsheng Chen[1,5,*]

[1]Department of Materials Science and Engineering, National University of Singapore, Singapore, 117575, Singapore

[2]Department of Electrical and Computer Engineering, National University of Singapore, Singapore, 117575, Singapore

[3]Centre for Micro- and Nano-Electronics (CMNE), School of Electrical and Electronic Engineering, Nanyang Technological University, 639798, Singapore,

[4]Beijing Superstring Academy of Memory Technology, Beijing, 100176, China

[5]Chongqing Research Institute, National University of Singapore, Chongqing, 401120, China

[†] These authors contributed equally to this work.

[*]Authors to whom correspondence should be addressed: lihh04@163.com; ebktay@ntu.edu.sg; msecj@nus.edu.sg;

Supplementary Note 1. Topological properties of Mn_3Sn film

Supplementary Figure 1a shows the x-ray diffraction (XRD) θ-2θ scans results of the 40 nm thick Mn_3Sn grown on Si substrate. Compared with pure Si substrate, a clear Mn_3Sn (0002) crystal peak is observed in the film sample. Furthermore, by SQUID measurement (Supplementary Figure 1b), Mn_3Sn film is proven to exhibit a tiny net magnetization, verifying the non-colinear antiferromagnet nature of our deposited film.

We then measure the angle-dependent planar Hall effect (PHE) and anisotropic magnetoresistance (AMR) to verify the existing chiral anomaly in our deposited Mn_3Sn. As shown in Supplementary Figure 1c, both curves under 9 T well correspond to the chiral anomaly behavior brought by the Weyl fermions. Additionally, anomalous Nernst effect (ANE) is another important evidence of the presence of Weyl fermions[1]. As shown in Supplementary Figure 1d, a sizable ANE voltage can be obtained in our Mn_3Sn films. We also demonstrate that the ANE voltage scales with the magnetization which is indicated by the AHE resistance.

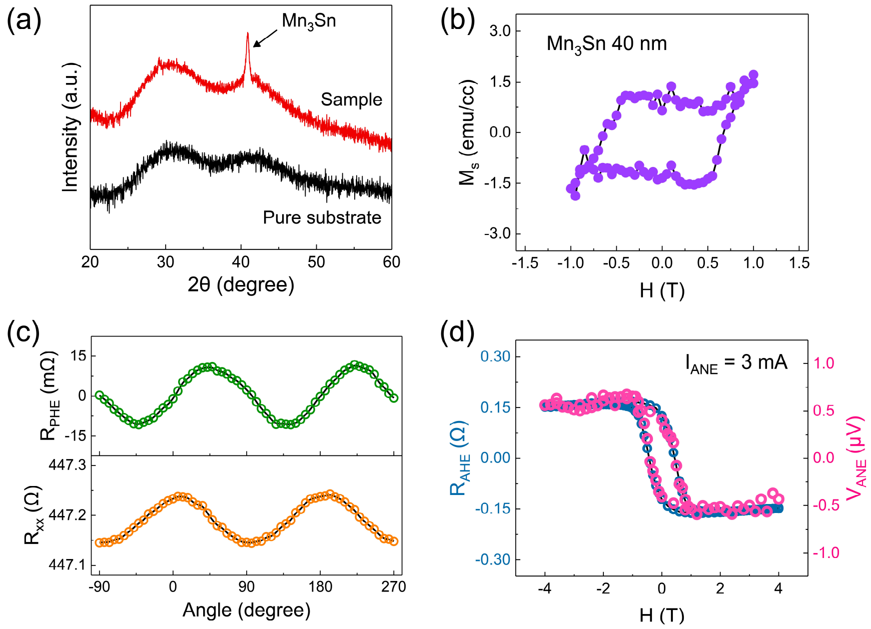

Supplementary Figure 1. a, XRD θ-2θ scans for pure Si substrate and Mn_3Sn film on Si substrate. A clear Mn_3Sn (0002) peak can be observed. **b**, Out-of-plane magnetic hysteresis loop of Mn_3Sn film. **c**, Angular dependence of longitudinal resistance R_{xx} and planar Hall resistance R_{PHE} under a large in-plane magnetic field of 9 T. **d**, The AHE and ANE loop in Mn_3Sn film.

Supplementary Note 2. Current-induced switching in Mn_3Sn/Pt bilayer

Here we investigate pure spin torque (ST) driven switching in Mn_3Sn/Pt bilayer, which serves as a comparison to the orbital torque (OT) driven switching results in Mn_3Sn/Pt/Mn trilayer. As shown in Supplementary Figure 2a, in the first sample where Pt thickness t_{Pt} is 0 nm, the current-induced switching is absent within the largest current range that we can apply without burning the device. It indicates that our deposited single Mn_3Sn film can't be switched by itself. Therefore, we verify that the switching driving forces mainly originate from the adjacent Pt or Pt/Mn layers. In Supplementary Figure 2b and 2c, we demonstrate that the critical switching current density J_c is reduced from 30 $\times 10^{10}$ A/m^2 to 20 $\times 10^{10}$ A/m^2 when t_{Pt} increases from 2 nm to 3 nm. This trend is distinct from the OT-driven J_c-t_{Pt} trend shown in Fig. 3a in main text and can be explained by the increase of effective spin Hall angle (SHA) in Pt.

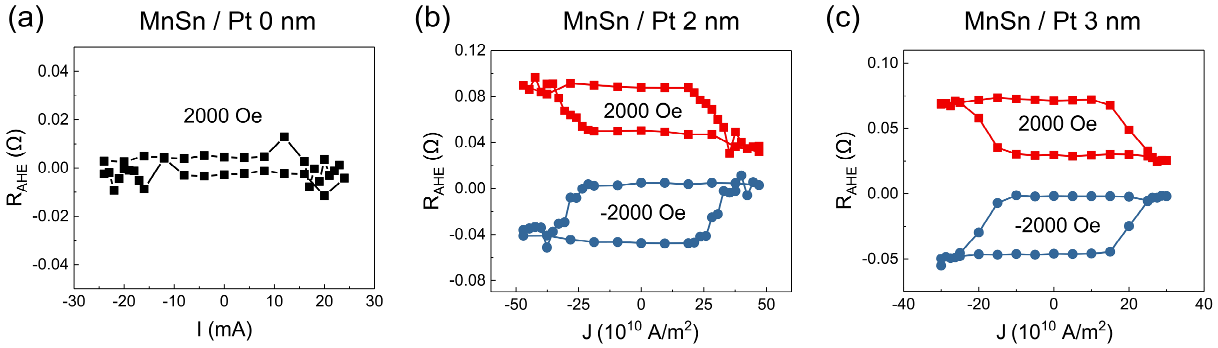

Supplementary Figure 2. Current-induced switching loops in samples **a**, single Mn_3Sn, **b**, Mn_3Sn/Pt(2 nm) and **c**, Mn_3Sn/Pt(3 nm).

Supplementary Note 3. Distinguishing orbital torque from spin torque in Cu/CuO_x

In main text, we demonstrated deterministic switching in Mn_3Sn/Cu/CuO_x heterostructure. Here, to better distinguish the switching driving force from Cu/CuO_x, we implemented second harmonics measurement in two samples Ni(5 nm)/Cu/CuO_x and Co(5 nm)/Cu/CuO_x. The following is our idea for clarifying the source of the torque: If the switching driving force is indeed the orbital torques, then these two samples should show distinct SOT effective fields, because Ni has stronger SOC than Co. However, if the driving force is the spin torques from interfacial Rashba SOC at the CuO_x/Cu interface or the bulk spin Hall effect of CuO_x as suggested by the reviewer, then these two samples should show similar SOT effective fields.

Supplementary Figure 3a shows the measurement setup where we rotate the magnetic field in the *xy* plane with an angle φ to the current direction. Supplementary Figure 3b and 3c show the first harmonic signal $R_{\omega}(\varphi)$ and second harmonic signal $R_{2\omega}(\varphi)$ in Ni/Cu/CuO_x sample under H = 0.5 T, respectively. The second harmonics signal can be fitted using the following equation suggested in [Nat. Commun., 12, 7111, 2021].

$$R_{2\omega}(\varphi) = \left(R_{\mathrm{AHE}}\frac{B_{\mathrm{DLT}}^{y}}{B_{\mathrm{eff}}} + R_{\nabla T}^{2\omega}\right)\cos\varphi + 2R_{\mathrm{PHE}}\frac{B_{\mathrm{FLT}}^{y}+B_{\mathrm{Oe}}}{B_{\mathrm{ext}}}(2\cos^{3}\varphi - \cos\varphi) -2R_{\mathrm{PHE}}\frac{B_{\mathrm{DLT}}^{z}}{B_{\mathrm{ext}}}\cos 2\varphi + R_{\mathrm{AHE}}\frac{B_{\mathrm{FLT}}^{z}}{B_{\mathrm{eff}}} \quad (1)$$

where B_{eff}, B_{DLT}^{y}, B_{FLT}^{y}, B_{DLT}^{z} and B_{FLT}^{z} are the effective field, y-polarized damping-like effective field, y-polarized field-like effective field, z-polarized damping-like effective field and z-polarized field-like effective field, respectively. Here, we mainly focus on B_{DLT}^{y}. Supplementary Figure 3d plots $R_{[\cos\varphi]}/R_{AHE}$ as a function of $1/B_{eff}$ in Ni/Cu/CuO_x and Co/Cu/CuO_x samples under the same applied current 3 mA. Here, $R_{[\cos\varphi]}$ is the cosφ component obtained by the fitting process, therefore the slope of the fitting curve in Supplementary Figure 3d indicates the value of B_{DLT}^{y}.

Apparently, Ni/Cu/CuO_x sample shows a very steep slope, while the slope of Co/Cu/CuO_x sample is negligible. This comparison reveals that Ni/Cu/CuO_x exhibits a much higher SOT

efficiency than Co/Cu/CuO_x, which unambiguously suggest that the main driving torque from Cu/CuO_x bilayer is the orbital torque.

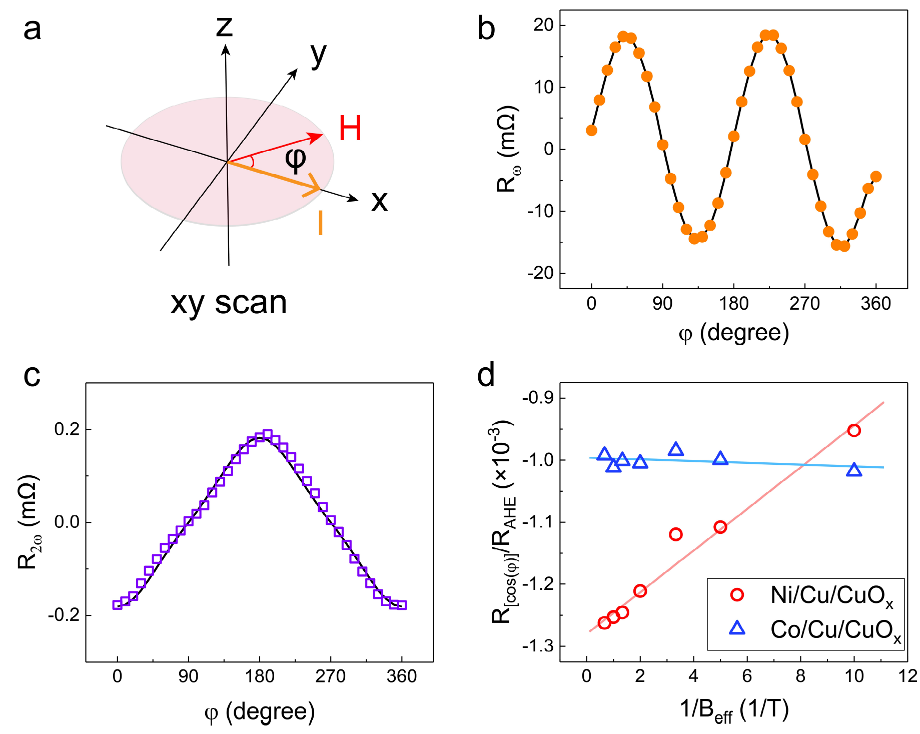

Supplementary Figure 3. a, Second harmonic measurement setup. **b** and **c**, first harmonic signal $R_\omega(\varphi)$ and second harmonic signal $R_{2\omega}(\varphi)$ as a function of φ in Ni/Cu/CuO_x sample under H = 0.5 T. **d**, $R_{[\cos\varphi]}/R_{AHE}$ as a function of 1/B_{eff} in Ni/Cu/CuO_x and Co/Cu/CuO_x samples.

Supplementary Note 4. Current-induced switching in Mn_3Sn/Pt/Mn trilayer

Supplementary Figure 4 illustrates the current-induced switching loops under an in-plane magnetic field of ±2000 Oe in Mn_3Sn/Pt(2 nm)/Mn(10 nm) and Mn_3Sn/Pt(6 nm)/Mn(10 nm) samples. We can conclude that the switching polarity of Mn_3Sn/Pt/Mn(10 nm) samples remain the same when varying the Pt thickness. It indicates that orbital torques from Mn layer are converted into spin torques which has the same sign as the spin torques generated by Pt itself.

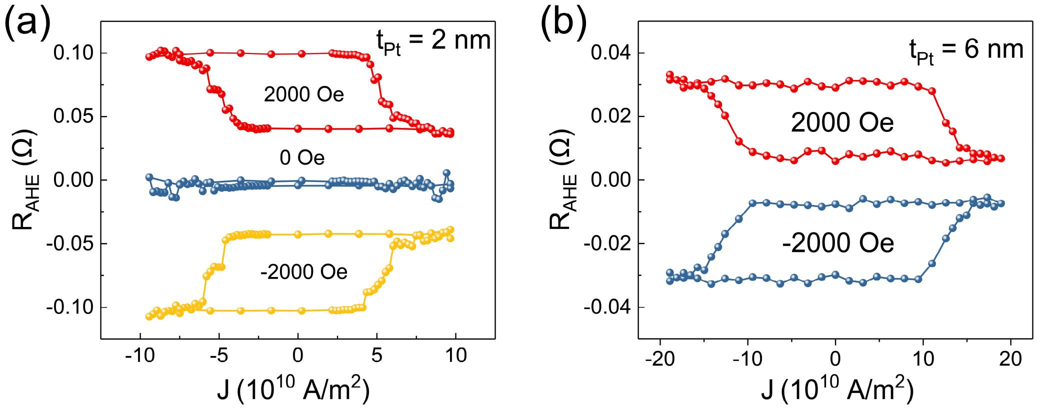

Supplementary Figure 4. Current-induced switching loops in **a**, Mn_3Sn/Pt(2 nm)/Mn(10 nm) and **b**, Mn_3Sn/Pt(6 nm)/Mn(10 nm).

Supplementary Note 5. Current-induced anomalous Nerst signal switching

From fundamental point of view, we also want to emphasize that our orbit-torque-based switching experiment not only switches the AFM order, but also impact the non-trivial topology in Mn_3Sn. To demonstrate this point, we did supplemental current-induced Anomalous Nerst effect (ANE) signal $V_{2\omega,ANE}$ switching experiment in Mn_3Sn/Pt/Mn device, since ANE has been proved as a typical transport characteristic of topology property in Mn_3Sn. The switching results are shown in Supplementary Figure 5. We can find that $V_{2\omega,ANE}$ switches simultaneously with the anomalous Hall signal R_ω, suggesting that non-trivial topology in Mn_3Sn can also be manipulated by the orbital torque.

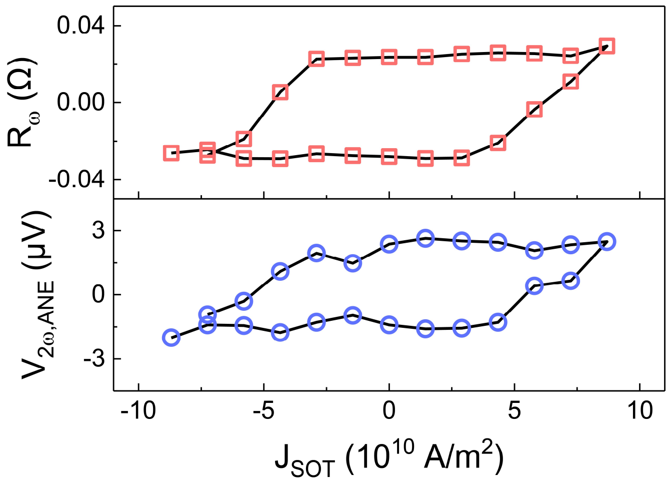

Supplementary Figure 5. Current-induced anomalous Hall signal and anomalous Nerst signal switching.

Supplementary Note 6. Field-induced multi-states in Mn_3Sn film

In main text, we successfully achieved multi-states in Mn_3Sn device by controlling the current pulse. In Supplementary Figure 6, we show that by limiting the largest magnetic field in the negative direction, multi-states can also be achieved in Mn_3Sn film. It verifies again the stability of the multi-states, which is of great significance to the realization of neuromorphic computing.

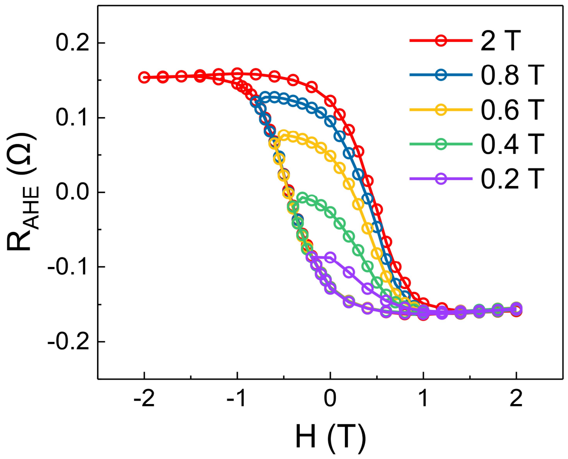

Supplementary Figure 6. Multi-state R_{AHE} loop in Mn_3Sn/Pt(2 nm)/Mn(10 nm) sample.

Supplementary Note 7. Effective spin Hall angle in Co/Pt/Mn system

To better quantify the amount of spin torques provided by Pt/Mn bilayer, we implemented spin-torque ferromagnetic resonance (ST-FMR) measurement in Co/Pt/Mn trilayer where the Co thickness is fixed to 5 nm. The measurement setup is shown in Supplementary Figure 7a. All the Co/Pt/Mn films were fabricated into 5-μm-wide stripes and connect to two metallic pads. The applied frequency varies from 3 GHz to 9 GHz. For instance, Supplementary Figure 7b plots the obtained ST-FMR signals V_{mix} as a function of applied in-plane magnetic field H_{ex} in the sample Co/Pt (2 nm)/Mn (10 nm). The spectrum can be well fitted to the sum of symmetric and anti-symmetric Lorentzian functions, which is written as[2]

$$V_{mix} = S\frac{\Delta^2}{\Delta^2+(H_{ex}-H_0)^2} + A\frac{\Delta(H_{ex}-H_0)}{\Delta^2+(H_{ex}-H_0)^2}, \quad (2)$$

where Δ, H_0, S and A are the linewidth (full width at half maximum), the resonant magnetic field, the symmetric Lorentzian coefficient and the anti-symmetric Lorentzian coefficient, respectively. Then, the effective SHA in the system can be calculated by[2]

$$SHA = \frac{S}{A}\frac{e\mu_0 M_s t d}{\hbar}[1 + (4\pi M_{eff}/H_0)], \quad (3)$$

where M_s, t, d and $4\pi M_{eff}$ are the saturated net magnetization of Co, the thickness of Co, the thickness of Pt/Mn bilayer and the demagnetization field of Co, respectively.

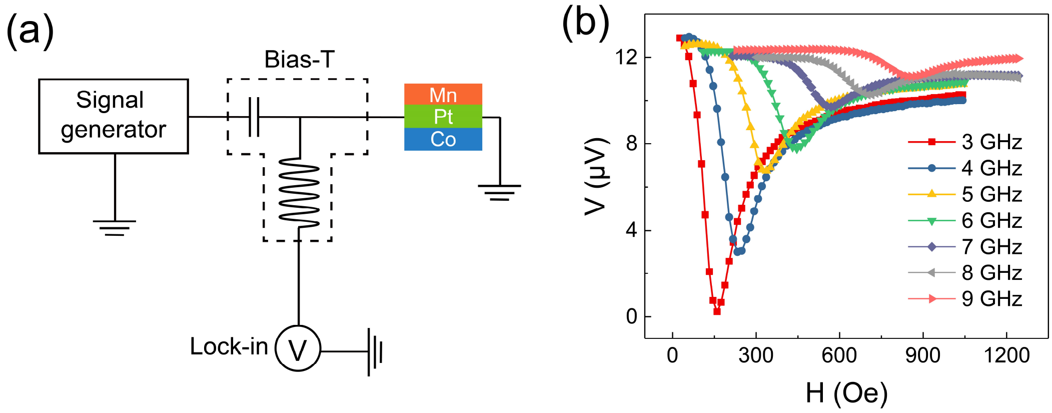

Supplementary Figure 7. a, ST-FMR measurement setup. **b**, ST-FMR signals versus H_{ex} under different frequency.

Supplementary Note 8. Detailed current distribution in Pt/Mn bilayers

In main text, we use the average current density in Pt/Mn bilayer. Here, we recalculated the current density distribution in Pt and Mn layer by the parallel shunting model. We determined the resistivity of Pt to be ~ 47 μΩ cm from a 2-nm-thick Pt/AlN device, while the resistivity of Mn was determined to be ~183 μΩ cm from a 10-nm-thick Mn/AlN device. We note that, when Pt thickness exceeds 5 nm, the resistivity of Pt would reduce a little and lead to a slightly larger calculated current density in Pt in the spin-torque-dominant zone. But it doesn't affect our core discussion at the small Pt thickness range (0-3 nm), i.e., the orbital-torque-dominant zone. The recalculated current density distributions in Pt and Mn corresponding to Fig. 3a and 3c in main text are plotted in Supplementary Figure 8a and 8b. We can find that those J_c trends show similar results compared to the trends we obtained by the average density. Therefore, our main conclusion is not affected.

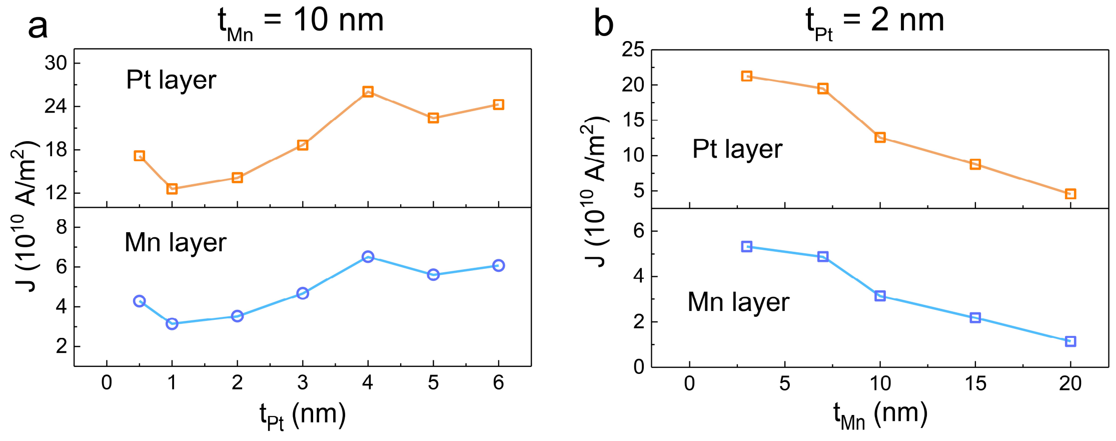

Supplementary Figure 8. a, Critical switching current distribution in Pt and Mn layers as a function of t_{Pt} with a fixed Mn thickness (t_{Mn} = 10 nm). **b**, Critical switching current distribution in Pt and Mn layers as a function of t_{Mn} with a fixed Pt thickness (t_{Pt} = 2 nm).

Supplementary Note 9. Discussion on power performance

In the main text, we have already reported that our orbital torque strategy can reduce J_c by more than one order of magnitude. Here, we would like to discuss a more realistic figure of merit for application, which is the switching power consumption *P*. The calculation is done by using the typical heat dissipation formula $P \propto {I_{Pt}}^2 R_{Pt} + {I_{Mn}}^2 R_{Mn}$. The detailed analysis of current distribution in Pt and Mn is shown in S8. We have done the performance comparison in two series of samples. In the first series (Supplementary Figure 9a), we fixed the thickness of Mn t_{Mn} to 10 nm and varied the thickness of Pt t_{Pt} from 0 nm to 6 nm. We normalized *P* with respect to the switching power of the sample with 6-nm-thick Pt, which can be considered as a totally spin-torque-dominant zone. In the second series (Supplementary Figure 9b), we fixed t_{Pt} to 2 nm and varied t_{Mn} from 0 nm to 20 nm. We normalized *P* with respect to the switching power of the sample with 0-nm-thick Mn, which also can be considered as a totally spin-torque-dominant zone. In both series, when the switching driving force is approaching the orbital-torque-dominant zone, *P* can be reduced by about one order of magnitude. This systematic comparison demonstrates the performance superiority of using orbital torque to switch Mn_3Sn.

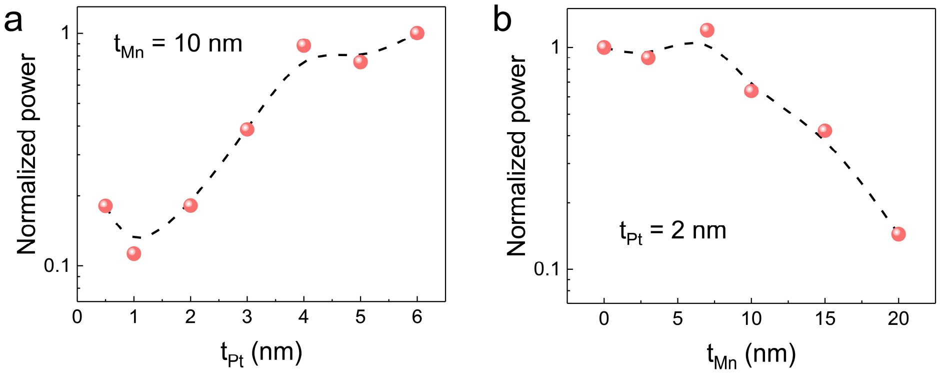

Supplementary Figure 9. a, Normalized power as a function of t_{Pt} with a fixed Mn thickness (t_{Mn} = 10 nm). **b**, Normalized power as a function of t_{Mn} with a fixed Pt thickness (t_{Pt} = 2 nm).

Supplementary Note 10. Harmonic measurement in Mn_3Sn devices

1) **First harmonics signal analysis**

Regarding the kagome plane in xz plane consists of three Mn atoms with the unit magnetic moment $\boldsymbol{m}_{(\mathrm{A,B,C})}$ (see illustration in Supplementary Figure 10) and if we consider the spin interaction microscopically, the total energy per unit volume can be expressed by[4]

$$\begin{aligned} &F(\boldsymbol{m}_A, \boldsymbol{m}_B, \boldsymbol{m}_C) = J(\boldsymbol{m}_A \cdot \boldsymbol{m}_B + \boldsymbol{m}_B \cdot \boldsymbol{m}_C + \boldsymbol{m}_C \cdot \boldsymbol{m}_A) \\ &-D\boldsymbol{e}_y \cdot (\boldsymbol{m}_A \times \boldsymbol{m}_B + \boldsymbol{m}_B \times \boldsymbol{m}_C + \boldsymbol{m}_C \times \boldsymbol{m}_A) \\ &-K_1((\boldsymbol{e}_A \cdot \boldsymbol{m}_A)^2 + (\boldsymbol{e}_B \cdot \boldsymbol{m}_B)^2 + (\boldsymbol{e}_C \cdot \boldsymbol{m}_C)^2) - \mu_0 M_0 \boldsymbol{H}(\boldsymbol{m}_A + \boldsymbol{m}_B + \boldsymbol{m}_C). \end{aligned} \tag{4}$$

where J and D are the exchange and DMI energy coefficients, respectively, M_0 is the magnetization of a single sublattice, and K_1 is the anisotropy energy coefficient. $\boldsymbol{e}_{(\mathrm{A,B,C})}$ are the single-ion uniaxial anisotropy axes for three sublattices, which correspond to the nearest neighbor Mn-Sn bond direction, and therefore can be written as $\boldsymbol{e}_A = -\frac{1}{2}\boldsymbol{e}_x + \frac{\sqrt{3}}{2}\boldsymbol{e}_z$, $\boldsymbol{e}_B = -\frac{1}{2}\boldsymbol{e}_x - \frac{\sqrt{3}}{2}\boldsymbol{e}_z$, $\boldsymbol{e}_C = \boldsymbol{e}_x$. In general, $J \gg D \gg K1$, which makes $\boldsymbol{e}_{(\mathrm{A,B,C})}$ forms 120° angle with respect to each other. The chirality is determined by the DMI. By defining $\varphi_{(\mathrm{A,B,C})}$ as the angle of $\boldsymbol{m}_{(\mathrm{A,B,C})}$ in the kagome plane, we have the relation $\boldsymbol{m}_{(\mathrm{A,B,C})} = \boldsymbol{e}_x cos\varphi_{(\mathrm{A,B,C})} + \boldsymbol{e}_z sin\varphi_{(\mathrm{A,B,C})}$.

We then define the cluster magnetic octupole moment $\boldsymbol{m}_{oct}$ by $\boldsymbol{m}_{oct} = \frac{1}{3}\boldsymbol{\mathcal{M}}_{xy}\left[\boldsymbol{R}\left(\frac{2\pi}{3}\right)\boldsymbol{m}_A + \boldsymbol{R}\left(-\frac{2\pi}{3}\right)\boldsymbol{m}_B + \boldsymbol{m}_C\right]$, where $\boldsymbol{R}$ is the rotation matrix, $\boldsymbol{R}\left(\pm\frac{2\pi}{3}\right)$ rotate $\boldsymbol{m}_A$ and $\boldsymbol{m}_B$ counterclockwise and clockwise by $2\pi/3$ in the kagome plane. $\boldsymbol{\mathcal{M}}_{xy}$ represents the mirror operation on the coordinates with respect to the xy plane, disregarding vector/pseudovector difference. Given any $\boldsymbol{m} = (m_x, m_y, m_z)$, $\boldsymbol{\mathcal{M}}_{xy}\boldsymbol{m} = (m_x, m_y, -m_z)$. In this definition, $|\boldsymbol{m}_{oct}| \approx 1$, because of the relatively rigid orientations of the three sublattices. Defining the azimuth angle of $\boldsymbol{m}_{oct}$ to be φ_{oct}, we have $\boldsymbol{m}_{\mathrm{oct}} = \boldsymbol{e}_x cos\varphi_{\mathrm{oct}} + \boldsymbol{e}_z sin\varphi_{\mathrm{oct}}$ and $\varphi_{\mathrm{oct}} = -\frac{\varphi_{\mathrm{A}}+\varphi_{\mathrm{B}}+\varphi_{\mathrm{C}}}{3}$.

Considering that our grown Mn_3Sn is polycrystalline, we should consider extra magnetic field anisotropy terms brought by lattice constant changes, etc. In most case, the lattice constant

changes in the in-plane direction, thus, the exchange constant J_{AB} is going to be different from J_{AC} and J_{BC}. Therefore, we change the exchange energy term to $J[(1-\Delta_{\mathrm{ex}})\boldsymbol{m}_A\cdot\boldsymbol{m}_B+\boldsymbol{m}_B\cdot\boldsymbol{m}_C+\boldsymbol{m}_C\cdot\boldsymbol{m}_A]$ with a factor Δ_{ex} to modify the change of J_{AB}. Then, we consider a strain-induced uniaxial magneto-crystalline anisotropy K_2 on all sublattices, with the corresponding easy axes are all along the x direction ($\boldsymbol{e}_C$). Then, the new microscopic magnetic energy can be written as[4]:

$$F(\boldsymbol{m}_A,\boldsymbol{m}_B,\boldsymbol{m}_C)=J\big((1-\Delta_{\mathrm{ex}})\boldsymbol{m}_A\cdot\boldsymbol{m}_B+\boldsymbol{m}_B\cdot\boldsymbol{m}_C+\boldsymbol{m}_C\cdot\boldsymbol{m}_A\big)-D\boldsymbol{e}_y\cdot(\boldsymbol{m}_A\times\boldsymbol{m}_B+\boldsymbol{m}_B\times\boldsymbol{m}_C+\boldsymbol{m}_C\times\boldsymbol{m}_A)-K_1((\boldsymbol{e}_A\cdot\boldsymbol{m}_A)^2+(\boldsymbol{e}_B\cdot\boldsymbol{m}_B)^2+(\boldsymbol{e}_C\cdot\boldsymbol{m}_C)^2)-K_2((\boldsymbol{e}_C\cdot\boldsymbol{m}_A)^2+(\boldsymbol{e}_C\cdot\boldsymbol{m}_B)^2+(\boldsymbol{e}_C\cdot\boldsymbol{m}_C)^2)-\mu_0M_0\boldsymbol{H}\cdot(\boldsymbol{m}_A+\boldsymbol{m}_B+\boldsymbol{m}_C). \quad (5)$$

Here we assume that the external field is applied within the kagome plane, then one can rewrite the magnetic energy as[4]:

$$\begin{aligned}F=&J\big((1-\Delta_{\mathrm{ex}})\cos(\varphi_B-\varphi_A)+\cos(\varphi_C-\varphi_B)+\cos(\varphi_A-\varphi_C)\big)+\\&D\big(\sin(\varphi_B-\varphi_A)+\sin(\varphi_C-\varphi_B)+\sin(\varphi_A-\varphi_C)\big)-K_1\Big(\cos^2\Big(\varphi_A-\tfrac{2\pi}{3}\Big)\\&+\cos^2\Big(\varphi_B+\tfrac{2\pi}{3}\Big)+\cos^2\varphi_C\Big)-K_2(\cos^2\varphi_A+\cos^2\varphi_B+\cos^2\varphi_C)\\&-\mu_0M_0H\big(\cos(\varphi_A-\beta)+\cos(\varphi_B-\beta)+\cos(\varphi_C-\beta)\big).\end{aligned} \quad (6)$$

The equilibrium condition is now $\frac{\partial F}{\partial\varphi_A}=\frac{\partial F}{\partial\varphi_B}=\frac{\partial F}{\partial\varphi_C}=0$. Note that an analytical solution for the equilibrium angle is too complicated to derive, one can determine $\varphi_{(\mathrm{A,B,C})}$ numerically, which further leads to φ_{oct} under any applied field condition. Using literature reported values of μ_0M_0 = 0.65 T, $K_1 = 6.3\times10^5$ J/m^3 , $J = 1.0\times10^8$ J/m^3 and substituting φ_{oct} into the anomalous Hall resistance R_ω expression $R_\omega=R_{AHE}sin\varphi_{oct}$, we find that the model fits the $R_\omega(\beta)$ data very well (see Fig. 3e), where the only two fitting parameters are K_2 and Δ_{ex}.

2) Second harmonics signal analysis

We assume that the SOT applies onto each sublattice separately and the applied field $\boldsymbol{H}$ and $\boldsymbol{m}_{(A,B,C)}$ remain in the kagome plane under zero applied current. Then, the magnetic dynamics can be described with the coupled Landau–Lifshitz–Gilbert equations:

$$\begin{aligned}\frac{\mathrm{d}\boldsymbol{m}_{(A,B,C)}}{\mathrm{d}t}=&-\gamma\mu_0\boldsymbol{m}_{(A,B,C)}\times\boldsymbol{H}_{\mathrm{eff}(A,B,C)}+\alpha\boldsymbol{m}_{(A,B,C)}\times\frac{\mathrm{d}\boldsymbol{m}_{(A,B,C)}}{\mathrm{d}t}\\&+\gamma\mu_0H_{\mathrm{DL}}\boldsymbol{m}_{(A,B,C)}\times\big(\boldsymbol{\sigma}\times\boldsymbol{m}_{(A,B,C)}\big).\end{aligned} \quad (7)$$

Previously we defined $\boldsymbol{m}_{oct} = \frac{1}{3}\boldsymbol{\mathcal{M}}_{xy}\left[\boldsymbol{R}\left(\frac{2\pi}{3}\right)\boldsymbol{m}_A + \boldsymbol{R}\left(-\frac{2\pi}{3}\right)\boldsymbol{m}_B + \boldsymbol{m}_C\right]$by rotation and mirror reflection transformations on $\boldsymbol{m}_{(\mathrm{A,B,C})}$. We would like to write out the equation that governs the dynamics of $\frac{\mathrm{d}\boldsymbol{m}_{\mathrm{oct}}}{\mathrm{d}t}$:

$$\begin{aligned}\frac{\mathrm{d}\boldsymbol{m}_{\mathrm{oct}}}{\mathrm{d}t} &= \frac{\mathrm{d}}{\mathrm{d}t}\left(\frac{1}{3}\boldsymbol{\mathcal{M}}_{xy}\left[\boldsymbol{R}\left(\frac{2\pi}{3}\right)\boldsymbol{m}_A + \boldsymbol{R}\left(-\frac{2\pi}{3}\right)\boldsymbol{m}_B + \boldsymbol{m}_C\right]\right) \\ &= \frac{1}{3}\boldsymbol{\mathcal{M}}_{xy}\left[\boldsymbol{R}\left(\frac{2\pi}{3}\right)\boldsymbol{\tau}_{\mathrm{M}}^{A} + \boldsymbol{R}\left(-\frac{2\pi}{3}\right)\boldsymbol{\tau}_{\mathrm{M}}^{B} + \boldsymbol{\tau}_{\mathrm{M}}^{C}\right] \\ &\quad + \frac{1}{3}\boldsymbol{\mathcal{M}}_{xy}\left[\boldsymbol{R}\left(\frac{2\pi}{3}\right)\boldsymbol{\tau}_{\mathrm{DL}}^{A} + \boldsymbol{R}\left(-\frac{2\pi}{3}\right)\boldsymbol{\tau}_{\mathrm{DL}}^{B} + \boldsymbol{\tau}_{\mathrm{DL}}^{C}\right],\end{aligned} \quad (8)$$

where the first and second terms are the torque from effective magnetic field $\boldsymbol{\tau}_{\mathrm{M}}^{oct}$ and damping-like (DL) spin torque $\boldsymbol{\tau}_{\mathrm{DL}}^{oct}$. Here we dropped the Gilbert damping torque for simplicity. In the dynamical switching process where $\frac{\mathrm{d}\boldsymbol{m}_{\mathrm{oct}}}{\mathrm{d}t} \neq \mathbf{0}$, spin torque can lead to a small out-of-kagome-plane tilt of $\boldsymbol{m}_{(\mathrm{A,B,C})}$ and induces fast precession of $\boldsymbol{m}_{(\mathrm{A,B,C})}$ and $\boldsymbol{m}_{oct}$. Since we use a low-frequency ac current, we here adopt the quasi-static limit of $\frac{d\boldsymbol{m}_{(\mathrm{A,B,C})}}{dt} \approx \mathbf{0}$ and $\frac{d\boldsymbol{m}_{oct}}{dt} \approx \mathbf{0}$.

According to the handedness anomaly in Ref. [4], we can get with $\boldsymbol{H}_{DL}^{oct} = -H_{DL}\boldsymbol{m}_{\mathrm{oct}} \times \boldsymbol{\sigma}$. In static case, the net torque on the octupole moment should be zero $\tau_{\mathrm{M}}^{\mathrm{oct}} + \tau_{\mathrm{DL}}^{\mathrm{oct}} = 0$, which leads to

$$\Delta\varphi_{\mathrm{oct}}(I) = \frac{-3H_{\mathrm{DL}}(I)}{H\left[\frac{K_1}{J}\cos\left(\beta-\varphi_{\mathrm{oct}}^0\right)-\frac{\lambda_1}{K_1}\cos\left(\beta+\varphi_{\mathrm{oct}}^0\right)-\frac{9\lambda_2}{K_1}\cos\left(\beta+3\varphi_{\mathrm{oct}}^0\right)\right]-\frac{4\lambda_1}{\mu_0 M_0}\cos\left(2\varphi_{\mathrm{oct}}^0\right)-\frac{16\lambda_2}{\mu_0 M_0}\cos\left(4\varphi_{\mathrm{oct}}^0\right)}. \quad (9)$$

Here we adopt the macroscopic anisotropy energy coefficient $\lambda_{1,2}$ in stead of the microscopic individual spin anisotropy parameter Δ_{ex} and K_2. Consequently, the second harmonic resistance can be obtained as $R_{2\omega} = \frac{1}{2}R_{AHE}cos\varphi_{oct}^0\Delta\varphi_{oct}(I_0)$ where I_0 is the peak ac current. With $\lambda_{1,2}$ obtained in the first harmonic measurement, we also well fit our $R_{2\omega}$ curve in Fig. 3f, with the only fitting parameter being H_{DL}. The effective spin Hall angle can then be calculated by $SHA = \frac{2e\mu_0(3M_0)tH_{DL}}{\hbar J}$, where M_0, t, $\hbar$ and J_{SOT} are the magnetization of a sublattice moment, the Mn_3Sn thickness, the reduced Planck constant and the average current density in the source layer, respectively.

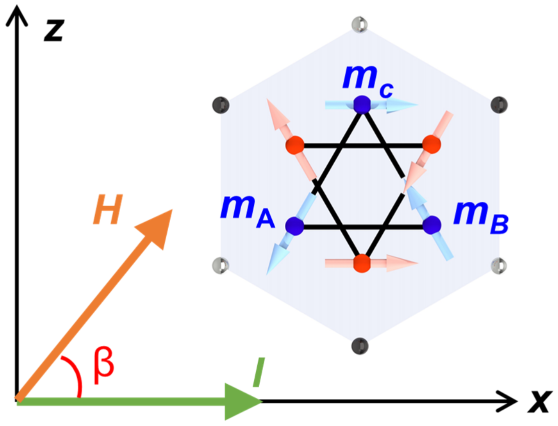

Supplementary Figure 10. Simple illustration of the described Mn_3Sn magnetic sublattices in harmonic measurement.

Supplementary Note 11. Discussion on the impact of linearity on recognition accuracy

We note that the current-induced memristor behavior in Mn_3Sn (Fig. 2e in main text) is only the precondition for realizing long-term depression (LTD) and the long-term potentiation (LTP) functions, and it cannot guarantee the achievement of LTD and LTP with high linearity. To prove this point, we also try to realize LTD and LTP functions in Mn_3Sn/Pt (5 nm) devices in which spin torque (ST) is the dominant switching driving force. As shown in Supplementary Figure 11a, the achieved nonlinearity (*NL*) of weight update is determined to be 0.686, which is much larger than that in both Mn_3Sn/Pt (2 nm)/Mn (10 nm) device (Fig. 4a in main text) and in the ideal device (Supplementary Figure 11b). This large NL value indicates a relatively bad linearity. While a good linearity is of great importance for constructing high-accuracy artificial neural network (ANN), if we use the Mn_3Sn/Pt device to construct an ANN, the corresponding learning accuracy rate is only 69.5% (see Fig. 4d in main text), which is much lower than our Mn_3Sn/Pt (2 nm)/Mn (10 nm) device in which orbital torque (OT) is the main switching driving force.

The relatively bad linearity in Mn_3Sn/Pt device can be explained by the following. According to our power consumption analysis, using Mn_3Sn/Pt device to do LTD and LTP would suffer from a much higher Joule heating effect. Therefore, a certain part of the Mn_3Sn will be switched in a thermally activated mode when the first several pulses are applied, causing an inhomogeneous switching in the pulse sequence, i.e., a worse linearity. As a comparison, using Mn_3Sn/Pt/Mn device to do LTD and LTP causes lower Joule heating. The switching process in the entire pulse sequence will be more homogeneous, inducing a better linearity. We thus conclude that orbital-torque-induced Mn_3Sn switching scheme is suitable for the realization of high accuracy ANN.

We here also discuss how we calculate that recognition accuracy. During learning training, structural similarity (SSIM) was employed to measure the difference between the learned image

(x) and input image (y). Herein, higher SSIM index corresponds to high recognition rate. The specific calculation equation can be expressed as follow:

$$SSIM(x,y) = f[l(x,y)^{\alpha},\ c(x,y)^{\beta},\ s(x,y)^{\gamma}], \tag{10}$$

where $l(x, y)$, $c(x, y)$, and $s(x, y)$ represent the image brightness, contrast, and structure, respectively. α, β, γ are parameter to modulate relative importance of different components[3]. In this work, all the parameters were set as 1. Therefore, the SSIM index was calculated with equation below:

$$SSIM(x,y) = f[l(x,y), c(x,y), s(x,y)]. \tag{11}$$

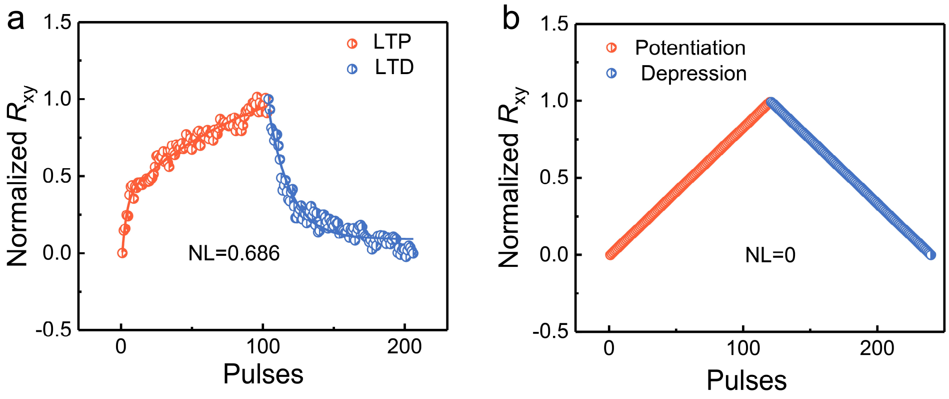

Supplementary Figure 11. a and **b**, LTD and LTP process in Mn_3Sn/Pt device and in an ideal device, respectively.

Supplementary References

1. Ikhlas, M. et al. Large anomalous Nernst effect at room temperature in a chiral antiferromagnet. *Nat. Phys.* **13**, 1085-1090 (2017).
2. Zhou, J. et al. Large spin-orbit torque efficiency enhanced by magnetic structure of collinear antiferromagnet IrMn. *Sci. Adv.* **5**, eaau6696 (2019).
3. Sheikh, H. R., Wang, Z., Bovik, A. & Simoncelli, E. Image quality assessment: from error visibility to structural similarity. *IEEE Trans. Image Process.* **13**, 600–612 (2004).
4. Yoon, J. Y. et al. Handedness anomaly in a non-collinear antiferromagnet under spin-orbit torque. *Nat. Mater.* **22**, 1106-1113 (2023).
